# Evaluation of the efficacy and safety of percutaneous transforaminal endoscopic discectomy for multi-segmental lumbar disc herniation

**DOI:** 10.12669/pjms.39.6.3399

**Published:** 2023

**Authors:** Ya-fei Zhao, Bin-wu Tian, Qiu-shuang Ma, Meng Zhang

**Affiliations:** 1 Ya-fei Zhao, Department of Orthopedics, Baoding No.1 Hospital of Traditional Chinese Medicine, Baoding 071000, Hebei, P.R. China; 2 Bin-wu Tian, Department of Orthopedics, Baoding No.1 Hospital of Traditional Chinese Medicine, Baoding 071000, Hebei, P.R. China; 3 Qiu-shuang Ma, Department of Orthopedics, Baoding First Central Hospital, Baoding 071000, Hebei, P.R. China; 4 Meng Zhang, Department of Orthopedics, Baoding No.1 Hospital of Traditional Chinese Medicine, Baoding 071000, Hebei, P.R. China

**Keywords:** Transforaminal endoscopy, Multi-segmental lumbar disc herniation, clinical efficacy, safety

## Abstract

**Objective:**

To investigate the clinical efficacy and safety of percutaneous transforaminal endoscopic discectomy (PTED) in the treatment of multi-segmental lumbar disc herniation (msLDH).

**Methods:**

From January 2021 to December 2021, 75 patients with msLDH admitted to Baoding No.1 Hospital of Traditional Chinese Medicine were selected and divided into PTED group (n=40) and posterior lumbar interbody fusion (PLIF) group (n=35) according to different surgical methods. The operative time, intraoperative blood loss, surgical complications, Oswestry disability index (ODI) and Japanese Orthopedic Association score (JOA) scores were compared between the two groups.

**Results:**

In the PTED group, the average operation time was 57.45±12.01minutes, and the average intraoperative blood loss was 50.57±16.69ml. There were three patients with surgical complications, including one case of hematoma, one case of aggravation of neurological symptoms and one case of new onset of neurological symptoms. In the PLIF group, there were 12 cases undergoing single-segment operation, 15 cases undergoing double-segment operation and 8 cases undergoing three-segment operation, the average operation time was 137.26±34.64minutes, and the average intraoperative blood loss was 456.06±33.06ml, there were four cases of wound fat liquefaction or delayed healing, two cases of hematoma, and three cases of exacerbation of original neurological symptoms or new neurological symptoms. At one month, six months, and one year of postoperative, the ODI and JOA scores of the two groups were significantly improved compared with those preoperative, and the ODI scores of the PTED group were better than those of the PLIF group (t=3.131, 2.263, 3.768, all P<0.05).

**Conclusion:**

The surgical effect of PTED in the treatment of LDH is similar to that of PLIF. However, PTED has the advantages of short operation time, less blood loss, fewer surgical complications, and high surgical safety. It is worthy of clinical promotion.

## INTRODUCTION

Lumbar disc herniation (LDH) is a common clinical disease, especially a common spinal surgery disease in the elderly, and its incidence has increased in recent years. The main clinical manifestations of LDH are low back pain, sciatica, and paresthesia in the innervation area.^1^,^2^ Data shows that approximately 70% of patients with LDH have symptoms of low back pain,, and about 1.2%-43% of patients have sciatica.^3^,^4^ The increase in axial load of intervertebral disc, combined with factors such as inflammatory factors, apoptosis and dehydration, leads to the degeneration of the intervertebral disc, which leads to the occurrence of LDH.^1^,^5^-^7^ In addition, lumbar disc degeneration often involves multiple segments, which can cause multi-segmental lumbar disc herniation(msLDH), The imaging data often shows that patients have msLDH, but in clinical practice, the symptoms of most patients are caused by a single protruding segment, which is what we call the “responsible segment”. In this study, patients in the percutaneous transforaminal endoscopic discectomy (PTED) group were performed with lumbar discography, and the “responsible segment” was determined by combining imaging data and clinical signs, and then PTED was performed. In the posterior lumbar interbody fusion (PLIF) group, single or multi-segment PLIF was performed according to preoperative imaging data and patient signs. The aim of this study was to explore the clinical efficacy and safety of PTED in the treatment of msLDH patients.

## METHODS

We selected 75 patients with multi-segment LDH admitted to Baoding No.1 Hospital of Traditional Chinese Medicine from January 2021 to December 2021, and divided them into PTED group and PLIF group based on different surgical methods. A total of 40 patients were enrolled in the PTED group, including 27 males and 13 females with an average age of 56.45 ± 9.81 (35-72) years old, and there were 35 patients in the PLIF group, including 20 males and 15 females with an average age of 55.63 ± 8.88 (34-75) years old. After the balance test, the general information of the two groups of patients is comparable. The surgery was performed by the same group of physicians, who had preoperative conversations with all patients and explained in detail the advantages and disadvantages of several surgical options to the patients. The surgical method is chosen by the patient and their family.

### Ethical Approval

The study was approved by the Institutional Ethics Committee of Baoding No.1 Hospital of Traditional Chinese Medicine, and written informed consent was obtained from all participants.

### Inclusion criteria:

- Age>18 years old.
- msLDH confirmed by CT and MRI imaging examinations, and all patients have typical clinical manifestations.
- Complete imaging data and follow-up data and follow-up time ≥ 1 year.
- Unsatisfactory after three months or more of conservative treatment.
- Through lumbar discography, clinical signs and imaging data, there is a clearly positioned “responsible segment” in the PTED group, And the responsible segment has not been treated with surgery.

### Exclusion criteria:

- Patients with single-segment LDH confirmed by imaging examination.
- Patients with previous lumbar surgery history.
- combined with other lumbar diseases, such as lumbar tuberculosis, spondylolisthesis, developmental deformity, scoliosis and lumbar fracture etc.
- Follow-up time <1 year.

### Lumbar discography

Patient were managed with lumbar discography in prone position. Skin disinfection and sheet laying were routinely carried out. First, the suspicious space was selected for lumbar discography. The puncture point was selected under the guidance of CT, local anesthesia was conducted with the use of 5 ml of 0.5% lidocaine, the puncture needle was slowly inserted into the intervertebral disc along the lateral border of the facet joint, when no liquid was withdrawn, 2ml of contrast agents (1.6 ml of iohexol + 0.4 ml of methylene blue) were injected, and the “responsible segment” was determined by observing the results of lumbar discography and judging the presence of reproduced pain of the original site or not.

### PTED

Patients were performed with PTED in prone position, and then local anesthesia was carried out. Skin disinfection and sheet laying were routinely conducted. The surgical segment was determined under fluoroscopy, and the puncture distance was also determined. The puncture needle was punctured to the superior articular process, and infiltration anesthesia was carried out on the facet joint. After the puncture needle reached the target point, the skin was cut by a sharp knife for about 0.7 CM, a guide wire was inserted to be gradually expanded through the sleeve, and the foraminoplasty operation was carried out by utilizing an abrasive drill. After placing the working channel, the protruded intervertebral disc and nucleus pulposus tissues were removed under an endoscope, nerve roots were fully decompressed until there was the visibility of pulsing of the nerve roots along with the pulse under the endoscope, and the skin was sutured after careful hemostasis with the radio-frequency electrode and covered with sterile dressing.

### PLIF

Patients were managed with PLIF in prone position, general anesthesia was carried out, and skin disinfection and sheet laying were routinely conducted. After the location of the surgical segment, a longitudinal incision was made through a posterior midline approach of spine, the skin, the fascia and the muscle of the patient was incised and stripped layer by layer to expose the vertebral plate. The pedicle screws were implanted through the vertebral pedicle of the vertebral body of the surgical segment, the internal fixation position was confirmed to be good under the C-arm fluoroscopy, two connecting rods were inserted, and the screws were locked after proper expansion. The spinous process, the bilateral vertebral plates and the inferior articular process were removed by a rongeur. The diseased annulus fibrosus of the intervertebral disc was cut by the sharp knife, the diseased intervertebral disc was taken out by a nucleus pulposus forceps, and the endplate was scraped off. The lateral recess and the nerve root canal were subjected to decompression. The intervertebral space was washed with physiological saline, the autogenous bone block or the allogeneic bone was implanted, and the screws were loosened and then re-locked after proper compression. The drainage tube was placed, and the wound was sutured layer by layer.

### Evaluation indexes:

###  Perioperative indicators

Operation time, intraoperative blood loss and complications (fat liquefaction or infection of surgical incision, hematoma formation, neurological deterioration, etc.).

The Japanese Orthopedic Association score (JOA) scoring system evaluated the spinal cord function preoperative, one week, one month, three months, six months and one year of postoperative. (Full score = 29 points).

The Oswestry Disability Index (ODI) scoring system was used to evaluate the degree of pain and dysfunction of patients preoperative, one week, one month, three months, six months and one year of postoperative.

### Statistical Analysis

SPSS 21.0 software was used for statistical analysis. The measurement data is expressed as`x±s, and the difference between the operation-related data of the two groups of patients is compared with the independent sample t test. The comparison of the parameters before and after the operation of the same patient is analyzed by variance analysis, the count data is expressed as n (%), and the c^2^ test was used for comparison, and P<0.05 was considered statistically significant.

## RESULTS

In the PTED group, the average operation time was 57.45±12.01 minutes, and the average intraoperative blood loss was 50.57±16.69 ml. There were three patients with surgical complications, including one case of hematoma, one case of aggravation of neurological symptoms, and one case of new onset of neurological symptoms.

Among the 35 patients in the PLIF group, there were 12 patients undergoing single-segment operation, 15 patients undergoing double-segment operation and eight patients undergoing three-segment operation. The average operation time was 137.26±34.64 minutes, and the average intraoperative blood loss was 456.06±33.06 ml. There were nine patients with surgical complications, including four patients of wound fat liquefaction or delayed healing, two patients of hematoma, three patients of aggravation of original neurological symptoms or new onset of neurological symptoms (Table-I).

**Table-I:** Comparison of operation situation between the two groups.

	Operation Time (Minute)	Intraoperative Blood Loss (Ml)	Surgical Complications (Case)
PTED Group	57.45 ± 12.01	50.57 ± 16.69	3
PLIF Group	137.26 ± 34.64	456.06 ± 33.06	9
Statistic Value	-12.964	-65.704	C^2^= 4.608
P Value	< 0.01	< 0.01	< 0.05

***Notes:*** PTED: Percutaneous Transforaminal Endoscopic Discectomy, PLIF: Posterior Lumbar Interbody Fusion.

There was no statistically significant difference in preoperative JOA score and ODI score between the two groups of patients (all P>0.05). However, at each follow-up time point postoperative, the JOA score and ODI score were significantly improved (all P<0.05), and the PTED group had better ODI scores than the PLIF group at one month, six months, and one year postoperative (t=3.131, 2.263, 3.768, all P<0.05) (Table-II).

**Table-II:** Comparison of ODI and JOA scores between the two groups.

	Before Operation	1 Week After Operation	1 Month After Operation	3 Months After Operation	6 Months After Operation	1 Year After Operation	F-Value	P-Value
***ODI***								
PTED Group	45.55± 9.72	55.45±8.78^a^	63.45±6.87^a^	69.90±11.30^a^	76.15±9.23^a^	83.20±11.49^a^	61.650	< 0.01
Open Surgery Group	45.43± 10.21	55.34±9.16^a^	58.00±8.20^a^	65.71± 12.94^a^	70.97±10.59^a^	73.31± 11.16^a^	339.439	< 0.01
T Value	0.053	0.052	3.131	1.496	2.263	3.768		
P Value	> 0.05	> 0.05	< 0.01	> 0.05	< 0.05	< 0.01		
***JOA***								
PTED Group	13.03± 3.41	16.45±3.68^a^	19.05±4.20^a^	21.20± 4.37^a^	22.38± 3.90^a^	24.10± 3.30^a^	725.174	< 0.01
Open Surgery	14.49± 3.64	16.03±4.43^a^	20.09±3.84^a^	21.31± 4.35^a^	22.31± 3.57^a^	22.91± 4.40^a^	361.161	< 0.01
T Value	-1.794	0.450	-1.109	-0.113	0.070	1.330		
P Value	> 0.05	> 0.05	> 0.05	> 0.05	> 0.05	> 0.05		

***Notes:*** JOA: Japanese Orthopedic Association, ODI: Oswestry Disability Index, PTED: Percutaneous Transforaminal Endoscopic Discectomy, A: The difference was statistically significant compared with preoperative data (P < 0.05).

## DISCUSSION

The common causes of intractable low back pain, radicular pain, and decreased mobility and disability in the elderly are lumbar degenerative disease and facet joint disease,^8^-^10^ because the elderly is more prone to lumbar degenerative disease, especially msLDH. msLDH is not only difficult to diagnose, but also more difficult to treat than single segment LDH, and the treatment method is controversial.^11^ Dural tear, nerve root injury complications such as residual nucleus pulposus and postoperative recurrence and re protrusion

For msLDH, in the past, clinical doctors often used multi-segment spinal open decompression and fusion surgery to ensure the surgical effect. Among them, PLIF, as one of the most classic surgical methods for multi-segment lumbar spine surgery, can provide good surgical vision, appropriate intervertebral height recovery and fusion, and biomechanical stability. elderly LDH patients themselves have severe degeneration of the intervertebral fibrous ring, and PLIF damages the posterior midline structure of the spine, such as the spinous process The vertebral arch, interspinous, and supraspinal ligaments further damage the stability of the intervertebral space, leading to a high probability of postoperative complications in PLIF. At the same time, the surgical time is long, the intraoperative blood loss is large, and it can also damage the soft tissue adjacent to the vertebral body and cause postoperative stubborn low back pain.^12^-^14^

At the same time, after PLIF intervertebral fusion, the range of motion of the lumbar fusion segment decreases, and overcompensation of adjacent segments may also lead to rapid degradation of adjacent intervertebral disc tissue, which is adjacent segment disease.^15^-^17^ With the rise and development of lumbar endoscopic technology, the development and application of endoscopic technology are more and more extensive. Spinal minimally invasive technology represented by endoscopic nucleus pulposus resection through Intervertebral foramen is increasingly used to treat elderly lumbar diseases. Research shows that minimally invasive procedure of spine has the following advantages:

- Short operation time and less blood loss during operation,
- Less damage to normal spinal structures,
- Facilitating postoperative rehabilitation and early mobilization without fusion surgery , to preserve the mobility of the lumbar spine.18-21

In a systematic review of percutaneous endoscopic and open surgery for lumbar disc herniation, Ruan Wenfeng and others found that there was no difference in functional improvement, complication rate and recurrence rate between PELD and open lumbar microdiscectomy, but through systematic comparison of seven studies, PELD significantly shortened the operation time and hospital stay.^22^

Existing studies have shown that the main clinical symptoms of patients with msLDH often originate from a single segment, also known as the “responsible segment”. The difficulty in surgical treatment of msLDH lies in determining the “responsible segment”, as its clinical symptoms are mixed and often inconsistent with imaging results. The determination of the “responsible segment” is more difficult than that of single segment LDH, Lumbar spine decompression, bone graft fusion, and internal fixation, represented by PLIF, tend to perform decompression and fusion on all spinal segments with abnormal imaging manifestations, which can easily lead to excessive decompression and significant damage to the spinal structure. Pre operative determination of the “responsible segment” in PELD and selective decompression of the “responsible segment” can achieve good surgical results.^11^,^23^ This study conducted lumbar disc imaging before endoscopic surgery, and determined the patient’s “responsible segment” based on the results of lumbar disc imaging, clinical symptoms, and imaging data.^24^-^26^ In addition, selective decompression of the “responsible segment” achieved the same functional results as open fusion surgery, reduced postoperative pain and dysfunction, and improved surgical safety.

### Limitations of this study

The PLIF used in this study for comparison with PTED has some common drawbacks of open surgery, and the specificity of the surgery can lead to more bleeding in patients. We look forward to further studies of percutaneous intervertebral foramen endoscopic discectomy and percutaneous endoscopic lumbar interbody fusion in the future, and attempt to evaluate the efficacy of these two surgical methods while comprehensively reducing complications.

### Authors’ Contributions:

**YZ & BWT:** Designed this study, prepared this manuscript, are responsible, accountable for the accuracy and integrity of the work.

**QSM:** Collected and analyzed clinical data.

**MZ:** Data analysis, significantly revised this manuscript.
